# Fibroblast growth factor-1 as a mediator of paracrine effects of canine adipose tissue-derived mesenchymal stem cells on in vitro-induced insulin resistance models

**DOI:** 10.1186/s12917-018-1671-1

**Published:** 2018-11-16

**Authors:** Hyeon-Jin Kim, Qiang Li, Woo-Jin Song, Hye-Mi Yang, Su-Yeon Kim, Sang-Chul Park, Jin-Ok Ahn, Hwa-Young Youn

**Affiliations:** 1 Department of Veterinary Internal Medicine, College of Veterinary Medicine, Seoul National University, Seoul, 08826 Republic of Korea; 2 Current Address: Department of Veterinary Internal Medicine, College of Veterinary Medicine, Kangwon National University, Chuncheon, 24341 Gangwondo Republic of Korea

**Keywords:** Fibroblast growth factor-1, Canine, Adipose tissue, Mesenchymal stem cells, Conditioned medium, Diabetes mellitus, Insulin resistance, In vitro, 3T3-L1

## Abstract

**Background:**

In the field of diabetes research, many studies on cell therapy have been conducted using mesenchymal stem cells. This research was intended to shed light on the influence of canine adipose-tissue-derived mesenchymal stem cell conditioned medium (cAT-MSC CM) on in vitro insulin resistance models that were induced in differentiated 3T3-L1 adipocytes and the possible mechanisms involved in the phenomenon.

**Results:**

Gene expression levels of insulin receptor substrate-1 (IRS-1) and glucose transporter type 4 (GLUT4) were used as indicators of insulin resistance. Relative protein expression levels of IRS-1 and GLUT4 were augmented in the cAT-MSC CM treatment group compared to insulin resistance models, indicating beneficial effects of cAT-MSC to DM, probably by actions of secreting factors. With reference to previous studies on fibroblast growth factor-1 (FGF1), we proposed FGF1 as a key contributing factor to the mechanism of action. We added anti-FGF1 neutralizing antibody to the CM-treated insulin resistance models. As a result, significantly diminished protein levels of IRS-1 and GLUT4 were observed, supporting our assumption. Similar results were observed in glucose uptake assay.

**Conclusions:**

Accordingly, this study advocated the potential of FGF-1 from cAT-MSC CM as an alternative insulin sensitizer and discovered a signalling factor associated with the paracrine effects of cAT-MSC.

**Electronic supplementary material:**

The online version of this article (10.1186/s12917-018-1671-1) contains supplementary material, which is available to authorized users.

## Background

Diabetes mellitus (DM) is an important endocrine disease that accounts for a serious proportion of small animal medicine. In dogs, most DM patients are known to be type 1 DM (T1DM), which is usually well managed by exogenous insulin supplements. However, canine DM with insulin resistance, ‘poorly controlled T1DM’, not only needs expensive treatment, but also has poor prognosis. Several reports advocate that concurrent diseases such as obesity and inflammatory diseases are associated with insulin resistance in human DM patients [1–4]. A study in dogs also asserts the obesity is a cause of insulin resistance [5, 6]. However, the exact pathophysiology of insulin resistance development is not fully understood. Although insulin sensitizers could be an option for the treatment of uncontrolled T1DM, their possible adverse effects such as weight gain, bone loss, and congestive heart failure encourage further effort to develop new strategy for insulin resistance [7].

Mesenchymal stem cells (MSCs) are multipotent stromal cells that have immunomodulatory and regenerative effects. Because they are relatively free from ethical issues, their therapeutic uses for various diseases including DM have been studied globally. Many studies have shown that they have advantageous effects in in vivo experiments using diabetic rodent models [8–13]. Until now, the known mechanisms of MSC actions when applied to DM are as follows: to differentiate directly into insulin-producing cells (IPCs), to regulate the immune system, and to secrete beneficial cytokines and growth factors [14]. In particular, the paracrine effects of MSCs are thought to enhance insulin sensitivity [15].

Fibroblast growth factor-1 (FGF1) which is a member of the FGF family has been known to play crucial role in glucose homeostasis [16]. Perry et al. reported that FGF1 and FGF19 improved glucose metabolism via down regulation of the hypothalamic-pituitary-adrenal axis [17]. It has been documented that exogenous recombinant FGF1 (rFGF1) improved insulin sensitivity as well as normalized blood glucose levels in diabetic mice models [18]. Recently, FGF1 is getting attention as a leading candidate of novel insulin sensitizer.

This study was designed to investigate the effects of canine adipose tissue-derived MSC-conditioned medium (cAT-MSC CM) on an in vitro induced insulin resistance model. To explore this, gene expression of markers related to glucose uptake were evaluated and the specific effective factors were discovered.

## Results

### Characterization of cAT-MSCs

**Fig. 1:**
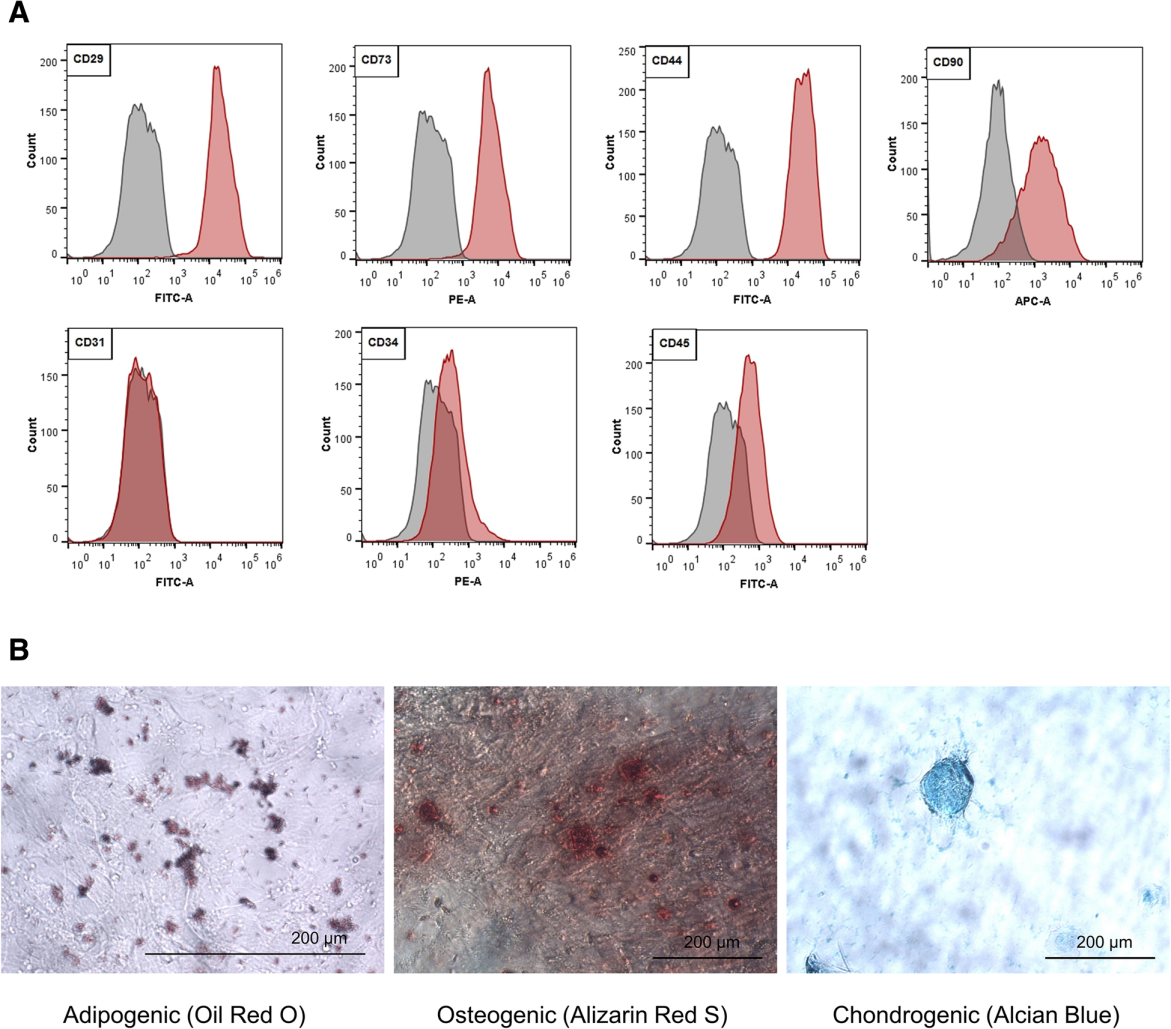
Flow cytometry and special staining characterized cAT-MSCs. **a** Immunophenotypic analysis was performed by flow cytometry using positive markers CD29, CD73, CD44, and CD90 and negative markers CD31, CD34, and CD45. **b** Adipogenic (Oil Red O staining), osteogenic (Alizarin Red S staining), and chondrogenic (Alcian Blue staining) differentiation abilities of cAT-MSCs (from left to right) were confirmed. Original magnification: 400x (left), 200x (middle, right)

Cells obtained from canine adipose tissue were characterized by their ability to express stem cell markers and to differentiate toward adipogenic, osteogenic, and chondrogenic lineages when cultured in media containing lineage-specific factors. The known MSC markers such as CD29, CD73, CD44, and CD90 were highly expressed by the cells. Negative markers such as CD31, CD34, and CD45 were not expressed (Fig. 1a). The multi-lineage plasticity of cAT-MSCs was confirmed by specific staining methods: Oil Red O staining, Alizarin Red S staining, and Alcian Blue staining, respectively (Fig. 1b).

### Differentiation into adipocytes and development of an insulin resistance model in 3T3-L1 adipocytes

**Fig. 2:**
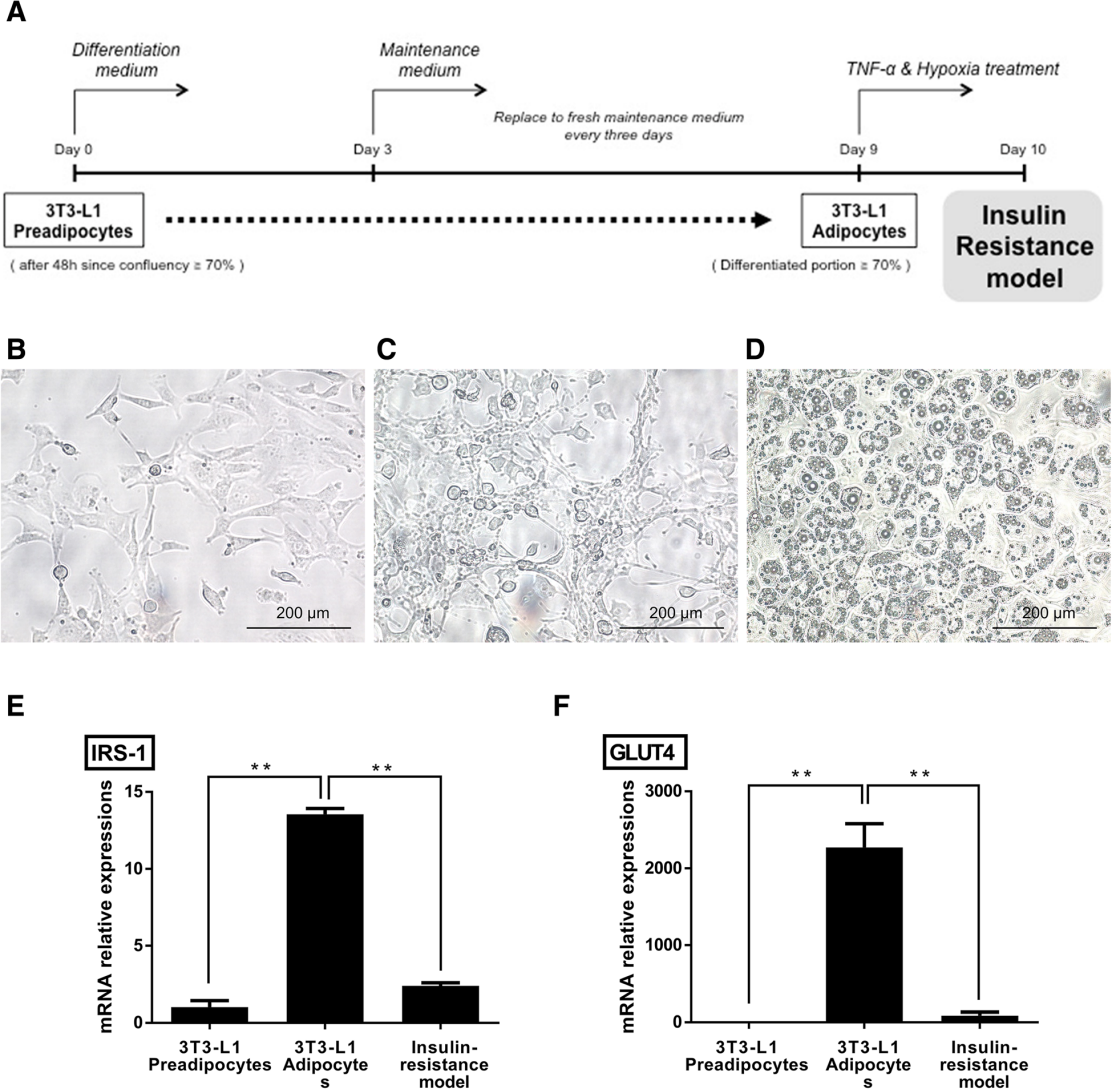
Insulin resistance was induced in 3T3-L1 adipocytes in vitro. **a** Adipocyte differentiation of 3T3-L1 cells and following induction of insulin resistance were summarized with the course of time. **b** Fibroblast-like 3T3-L1 pre-adipocytes at day 0. **c** Differentiating 3T3-L1 cells at day 3. **d** Differentiated 3T3-L1 adipocytes containing cytoplasmic lipid droplets at day 9. Original magnification: 200x. **e**, **f** Alterations in mRNA expression levels of IRS-1 and GLUT4 during the differentiation and insulin resistance induction were shown. Results are presented as mean ± SD obtained from three independent experiments *P < 0.05, **P < 0.01

All processes of differentiation and induction of insulin resistance in the 3T3-L1 are depicted schematically in Fig. 2a. During differentiation, 3T3-L1 pre-adipocytes transformed from a fibroblast-like appearance to an adipocyte-like appearance and cytoplasmic lipid droplet accumulation became remarkable (Fig. 2b-d). After over 70% of 3T3-L1 cells differentiated, cells were treated with TNF-α and incubated in hypoxic conditions for 24 h to induce insulin resistance. The mRNA expression levels of IRS-1 and GLUT4 were compared as markers of insulin resistance. As a result, both IRS-1 and GLUT4 mRNA levels showed similar tendencies through the differentiation to insulin resistance induction, but the degree of alterations was greater in GLUT4. The mRNA expression levels of insulin resistance markers were remarkably increased during the differentiation, while they were markedly decreased in the insulin resistance model (Fig. 2e, f).

### Effects of cAT-MSC CM treatment on IRS-1 and GLUT4 expressions in the insulin resistance model

**Fig. 3:**
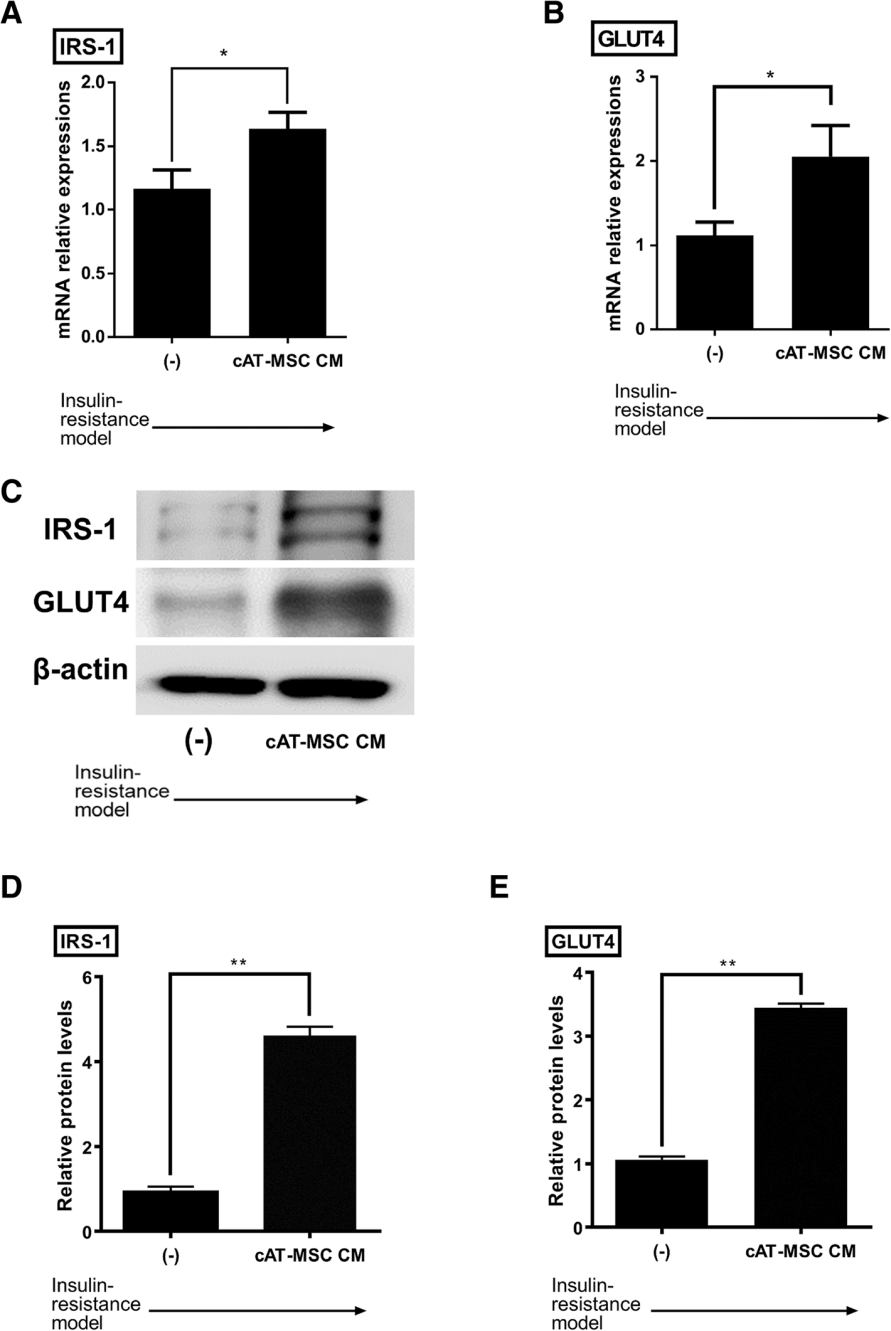
Treatment of cAT-MSC CM affected gene expressions of IRS-1 and GLUT4 in 3T3-L1 insulin resistance models. **a, b** The mRNA expression levels of IRS-1 and GLUT4 were elevated significantly in CM-treated insulin resistance models. **c** Western blot. **d, e** Up regulation of IRS-1 and GLUT4 protein expression was observed in CM-treated insulin resistance models. (−): insulin resistance models of 3T3-L1 cells, cAT-MSC CM: cAT-MSC CM-treated insulin resistance models of 3T3-L1 cells. Results are presented as mean ± SD obtained from three independent experiments *P < 0.05, **P < 0.01

To evaluate the therapeutic effects of cAT-MSC CM on the insulin resistance model, the expression levels of IRS-1 and GLUT4 were examined by qRT-PCR and western blot analysis. Both IRS-1 and GLUT-4 mRNA expression levels were significantly increased after cAT-MSC CM treatment in the insulin resistance model (Fig. 3a, b). Similar results were observed in protein analysis (Fig. 3c-e, Additional file 1: Figure S1).

### Reduced therapeutic effects of cAT-MSC CM with anti-FGF1 neutralizing antibody treatment

**Fig. 4:**
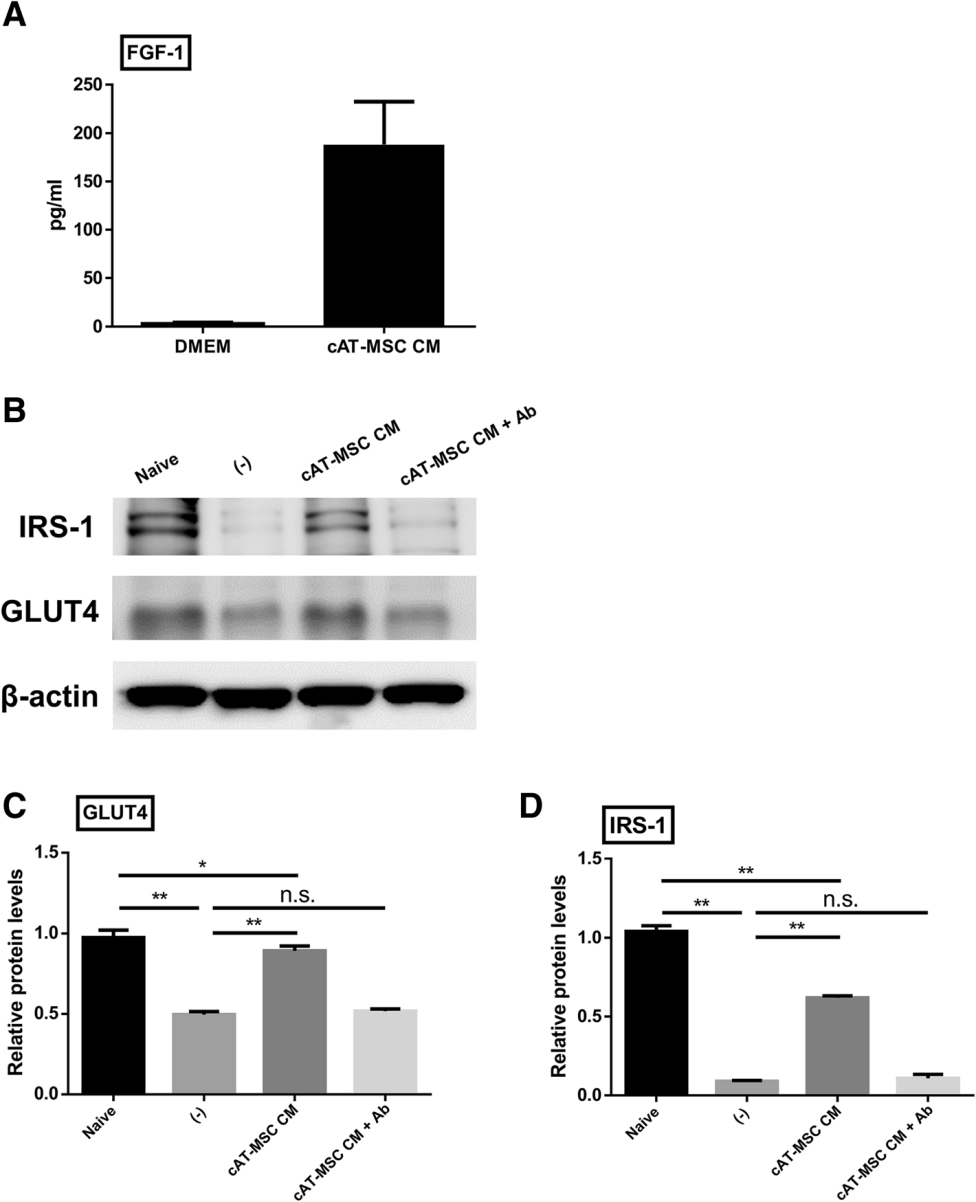
Protein expressions of IRS-1 and GLUT4 were down regulated in differentiated 3T3-L1 adipocytes after blocking of FGF1 function. **a** FGF-1 concentrations in cAT-MSC CM and DMEM were measured by ELISA assay. **b** Protein expression levels of GLUT4 and IRS-1 were analysed by western blot. **c, d** Relative protein levels of IRS-1 and GLUT4 were lower in the insulin resistance models treated with both cAT-MSC CM and anti-FGF1 antibody than the models treated with cAT-MSC CM alone. Naïve: differentiated 3T3-L1 cells, (−): insulin resistance models of 3T3-L1 cells, cAT-MSC CM: cAT-MSC CM-treated insulin resistance models of 3T3-L1 cells, cAT-MSC CM + Ab: cAT-MSC CM- and anti-FGF1 antibody-treated insulin resistance models. Results are presented as mean ± SD obtained from three independent experiments *P < 0.05, **P < 0.01

**Fig. 5:**
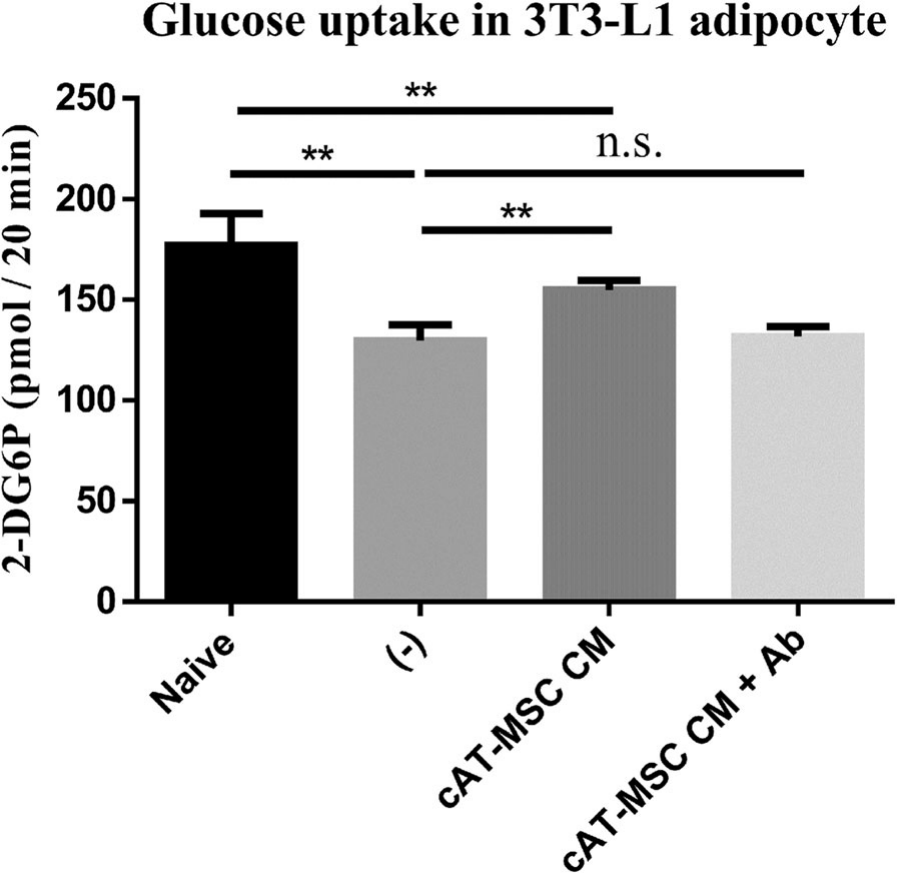
Effect of FGF-1 on 2-DG glucose uptake in insulin resistance models of 3T3-L1 cells. Naïve: differentiated 3T3-L1 cells, (−): insulin resistance models of 3T3-L1 cells, cAT-MSC CM: cAT-MSC CM-treated insulin resistance models of 3T3-L1 cells, cAT-MSC CM + Ab: cAT-MSC CM- and anti-FGF1 antibody-treated insulin resistance models. Results are presented as mean ± SD obtained from three independent experiments *P < 0.05, **P < 0.01

We suspected that FGF1 in the cAT-MSC CM played an important role in the alterations in the expression levels of IRS-1 and GLUT4. To verify our hypothesis, we measured FGF-1 concentrations in DMEM and cAT-MSC CM by ELISA. FGF-1 concentrations were significantly increased in cAT-MSC CM compared to DMEM (Fig. 4a). Moreover, added an anti-FGF1 neutralizing antibody to the cAT-MSC CM treated insulin resistance model. By measuring the protein levels of insulin resistance markers and evaluating glucose uptake abilities, we confirmed that the improvement of expression levels of IRS-1, GLUT4 (Fig. 4b-d), and glucose uptake ability (Fig. 5, Additional file 1: Figure S1) were significantly reduced after anti-FGF1 neutralizing antibody treatment compare to cAT-MSC CM treated group.

## Discussion

There have been doubts that exogenous insulin supplements for poorly controlled T1DM or T2DM were not enough to maintain the patient in a good condition. In this regard, researches into cell therapy using MSCs have been actively conducted. Mechanisms of their therapeutic effects to DM may be explained through various molecular processes. MSCs have the ability to directly differentiate into IPCs [19–21] and regulate the immune system to protect pancreatic islet cells from further destruction [22–26]. In addition, they possess powerful paracrine effects via secretion of a variety of cytokines and growth factors, enabling them to have anti-inflammatory effects and regulate insulin signalling and resulting in the improvement of insulin sensitivity [15, 27–29]. Several insulin sensitizers (such as biguanides and thiazolidinediones) were considered as therapeutics for DM with insulin resistance. However, long-term medication with these drugs can cause various adverse effects, which highlights the necessity for a new strategy.

In these experiments, we used 3T3-L1 adipocytes to develop an in vitro insulin resistance model. There have been diverse studies into inducing insulin resistance in 3T3-L1 cell lines. For example, various agents including TNF-α, Interleukin-1 (IL-1), IL-6, free fatty acids, dexamethasone, and high insulin were used to make models of insulin resistance in 3T3-L1 adipocytes [30–35]. Lo et al. compared transcriptome analysis data of in vitro insulin resistance models and in vivo diet-induced obese mouse models. Considering their finding that the TNF-α and hypoxia co-treatment model most closely resembled in vivo alterations [36], we employed this model in our experiment. For the purpose of monitoring insulin resistance, we selected IRS-1 and GLUT4 as markers of glucose uptake ability [37, 38]. IRS-1 is a signalling protein that plays an important role at the initial part of insulin signalling pathway [39]. GLUT4 is a transporter protein that is mainly expressed in the skeletal muscle, adipose tissue, and heart [40]. Its up regulation and translocation to the cell membranes are thought to be critical steps of the insulin responsive increment of glucose uptake [41].

According to early studies, the effectiveness of MSC in DM originated from the potential to trans-differentiation towards IPCs [42, 43]. However, these theories were unable to give a good explanation for the disappointing results of in vivo cell tracking studies [44, 45]. Recent studies suggested that the secretory capacity of MSCs would play a crucial role for the therapeutic effects. Gao et al. isolated and injured pancreatic islets in vitro and then assessed islet regeneration after the treatment with MSC CM. They found that both β cell replication and islet progenitor differentiation were promoted and the PI3K / Akt signal pathway was involved [46]. Another study revealed that AT-MSC CM enhanced glucose uptake in 3T3-L1 and C2C12 insulin resistance models [47]. We also utilized cAT-MSC CM to explore the paracrine effect of cAT-MSC in our experiments.

FGF family is a signalling protein group composed of 18 members mediating a variety of biological functions in cell proliferation and developmental processes. Each family member performs unique actions by binding to FGF receptors, which results in activation of intracellular downstream cascades [48]. FGF1, identified as a regulator of adipose tissue remodelling and metabolic homeostasis, is a downstream molecule of peroxisome proliferator-activated receptor-γ (PPAR-γ), which is a target of the thiazolidinedione class of insulin sensitizers [49]. It has been established that rFGF1 injection normalized glucose levels and enhanced insulin sensitivity in diabetic mice. What was noteworthy is that the effect was insulin-dependent. Moreover, rFGF1 did not induce side effects that could be incurred by chronic use of traditional insulin sensitizers. Furthermore, genetically modified rFGF1 lacking the mitogen properties of rFGF1 had similar glucose lowering effects to some extent [18], intensifying the possibility of rFGF1 as a substitute of conventional insulin sensitizers. Based on the fact that MSC CM contains considerable amounts of FGF1 [50], we attempted to find out whether FGF1 from cAT-MSC CM was contributed to the advantageous effects of cAT-MSC CM on insulin resistant models.

In the present study, we evaluated gene expression levels of IRS-1 and GLUT4, which were considered as markers of insulin responsive glucose uptake. It was shown that reduced expression levels of these markers in 3T3-L1 insulin resistance models were restored after the treatment of cAT-MSC CM. This finding was consistent with previous research that suggested that MSC could improve insulin resistance through the paracrine signalling of various cytokines and growth factors [47]. Since we suspected FGF1 would have a role in this effect, we measured FGF1 concentrations in cAT-MSC CM and added an anti-FGF1 neutralizing antibody into CM-treated 3T3-L1 insulin resistance models and assessed alterations of glucose uptake levels. The anti-FGF1 antibody group represented diminished expression levels of insulin resistance markers in comparison to the CM-treated group. This information led to the inference that FGF1 included in cAT-MSC CM is one of the mediators of the signalling pathways that induces therapeutic effects. Although we could not exclude the possibility that various cytokines and growth factors abundantly contained in cAT-MSC CM (other than FGF1) could affect the alterations in expression levels, it is worth mentioning that this study is the first work that revealed FGF1 as a specific mediator of insulin-sensitizing effects of cAT-MSC CM in DM.

## Conclusions

We identified that the induced insulin resistance in 3T3-L1 cells was ameliorated in the presence of cAT-MSC CM by measuring mRNA and protein expression levels of IRS-1 and GLUT4. Additional data showed that the improvement was inhibited by the addition of anti-FGF1 neutralizing antibody, suggesting that FGF1 would act as a mediator of the beneficial effects of cAT-MSC CM. It is noteworthy that the present study is the first to reveal a specific functioning component of MSC CM in in vitro insulin resistance models, and further studies to find out other effective factors of MSC CM will be necessary.

## Methods

### Cell culture and characterization

Adipose tissue was obtained from three healthy dogs (1-year-old) during ovariohysterectomy at the Seoul National University Veterinary Medicine Teaching Hospital (SNU VMTH). The owners were provided an informed, written consent for research use. The procedure was also approved by the Institutional Animal Care and Use Committee (IACUC) of SNU and the protocol was performed in accordance with the approved guidelines. Canine adipose tissue-derived mesenchymal stem cells (cAT-MSCs) were isolated and cultured as previously described [51]. Before their use in this study, cells were characterized by their ability to express several stem cell markers using flow cytometry. Cells were suspended in 30 μl DPBS and 3 μl monoclonal antibodies against the following proteins: cluster of differentiation (CD) 29-Fluorescein isothiocyanate (FITC), CD31-FITC, CD34-phycoerythrin (PE), CD73-PE (BD Biosciences, Franklin Lakes, NJ, USA), CD44-FITC, CD45-FITC, and CD90-allophycocyanin (APC) (eBiosciences, San Diego, CA, USA)-conjugated antibodies. Non-stained cells were used as controls for autofluorescence. Cells were analysed by a BD FACSAria II system (BD Biosciences). Cellular differentiation was confirmed using PRIME-XV® Chondrogenic Differentiation Xeno-Free Serum-Free Medium (XSFM), PRIME-XV® Osteogenic Differentiation Serum-Free Medium (SFM), and PRIME-XV® Adipogenic Differentiation SFM (all from Irvine Scientific, Santa Ana, CA, USA) according to the manufacturer’s instructions followed by Alcian Blue staining, Alizarin Red staining, and Oil Red O staining, respectively.

### In vitro cellular insulin resistance models

The in vitro induced insulin resistance model was developed in differentiated 3T3-L1 adipocytes. Murine 3T3-L1 cells were purchased from the Korean Cell Line Bank (Seoul, Korea). 3T3-L1 pre-adipocytes were differentiated using 3T3-L1 Differentiation Kit (Sigma-Aldrich) and all the differentiation procedures performed following the manufacturer’s instructions. In brief, 3 × 10^5^ cells were seeded in 6 well cell culture plate (SPL Life Science, Pocheon, Korea) and reached 70%, induction of insulin resistance was initiated. Cells were washed with phosphate-buffered saline (PBS; PAN Biotech, Aidenbach, Germany) and changed to serum-free Dulbecco’s modified Eagle’s medium: Nutrient Mixture F-12 (1:1) (DMEM / F12 (1:1); PAN Biotech). Insulin resistance was induced with treatment with both 40 ng/mL of tumour necrosis factor-α (TNF-α; PeproTech, Rocky Hill, NJ, USA) and 1% oxygen for 24 h. For hypoxic incubation, cells were placed in a hypoxic incubator (ViVAGEN, Sungnam, Korea) with the conditions of 1% O_2_ at 37 °C.

### Preparation of cAT-MSC-conditioned medium (CM), and anti-fibroblast growth factor-1 (FGF1) antibody treatment

cAT-MSCs (3 × 10^5^ cells / well) were seeded in 6-well plates and cultured in 3 mL of DMEM medium containing 2% fetal bovine serum (FBS; PAN Biotech) for 3 days, yielding conditioned medium. After 3 days, conditioned medium was harvested and centrifuged at 850 rpm for 3 min to remove cellular debris. After centrifugation, the supernatant was transferred to a conical tube and stored at − 80 °C until use. In addition, the concentration of FGF-1 in the cAT-MSC CM were determined by Fibroblast Growth Factor 1 ELISA Kit (Mybiosource, San Diego, CA, USA) according to the manufacturer’s instructions. Anti-FGF1 neutralizing antibody (Abcam, Cambridge, MA, USA) was added to final concentrations of 0.9 μg / ml of culture medium.

### RNA extraction, cDNA synthesis, and quantitative reverse-transcription PCR

Total RNA was extracted from all groups using the Easy-BLUE Total RNA Extraction kit (Intron Biotechnology, Seongnam, Korea) according to the manufacturer’s instructions. cDNA was synthesized from 1 μg of total RNA with LaboPass M-MuLV Reverse Transcriptase (Cosmo Genetech, Seoul, Korea) and the samples were analyzed in triplicates using AMPIGENE qPCR Green Mix Hi-ROX with SYBR Green dye (Enzo Life Sciences, Farmingdale, NY, USA). The expressions of target genes were analyzed according to the 2^−ΔΔ/Cts^ method and normalized to mRNA levels of glyceraldehyde-3-phosphate dehydrogenase (GAPDH). Primer sequences used in this study are listed in Table 1.

**Table 1:** List of primer for qRT-PCR

Gene	Forward (5′-3′)	Reverse (5′-3′)
GAPDH	AGTATGTCGTGGAGTCTACTGGTGT	AGTGAGTTGTCATATTTCTCGTGGT
GLUT4	CCCAGTGAGTCTGTCATCTAGTAGT	GGACTAGAACCATACTCATCAGAAG
IRS-1	GAACACTGGTCCTAGCTGTATTCTC	GTAGCTCTGTTCAATCACCTTCTGT

### Western blot analysis

Total proteins were extracted from all cell groups using PRO-PREP Protein Extraction Solution (Intron Biotechnology) according to the manufacturer’s instructions. The concentrations of the protein samples were measured using the Bio-Rad DC Protein Assay Kit (Bio-Rad, Hercules, CA, USA). The 30 μg of proteins were loaded and separated by sodium dodecyl sulphate-polyacrylamide gel electrophoresis and transferred to polyvinylidene difluoride membranes (Millipore, Billerica, MA, USA). The membranes were blocked by 5% non-fat dry milk in Tris-buffered saline containing 0.1% Tween 20 and incubated with primary antibodies against insulin receptor substrate 1 (IRS-1, 1:500; Abcam) and glucose transporter type 4 (GLUT4, 1:500; Santa Cruz Biotechnology, Santa Cruz, CA, USA) at 4 °C overnight. The membranes were incubated with secondary antibodies at room temperature for 1 h. The immunoreactive bands were visualised using enhanced chemiluminescence (Advansta, Menlo Park, CA, USA) and normalised to β-actin levels (Santa Cruz Biotechnology).

### 2-Deoxyglucose (2-DG) uptake assay

The differentiated 3T3-L1 adipocytes were treated with TNF-α and incubated in hypoxia for 24 h. After that, the 2-DG concentration was measured by Glucose Uptake Assay Kit (Abcam) according to the manufacturer’s instructions. Measurements were performed at least three replicates and then averaged.

### Statistical analysis

Data are shown as mean ± standard deviation. Statistical comparisons between groups were made with use of one-way ANOVA and an unpaired Student’s t test using the GraphPad Prism v.6.01 software (GraphPad Inc., La Jolla, CA, USA). *P* value of < 0.05 was considered statistically significant.

## Additional file

Additional file 1: **Figure S1.** Original western blot image. (PPTX 444 kb)
