# Supplementary material for: Fibroblast growth factor-1 as a mediator of paracrine effects of canine adipose tissue-derived mesenchymal stem cells on in vitro-induced insulin resistance models
Source: BMC Vet Res. 2018 Nov 16;14:351. doi: 10.1186/s12917-018-1671-1 (PMC6240186; doi:10.1186/s12917-018-1671-1)
Supplement: Supplementary file 1 — Figure S1. Original western blot image. (PPTX 444 kb) [file 12917_2018_1671_MOESM1_ESM.pptx]

## Slide 1
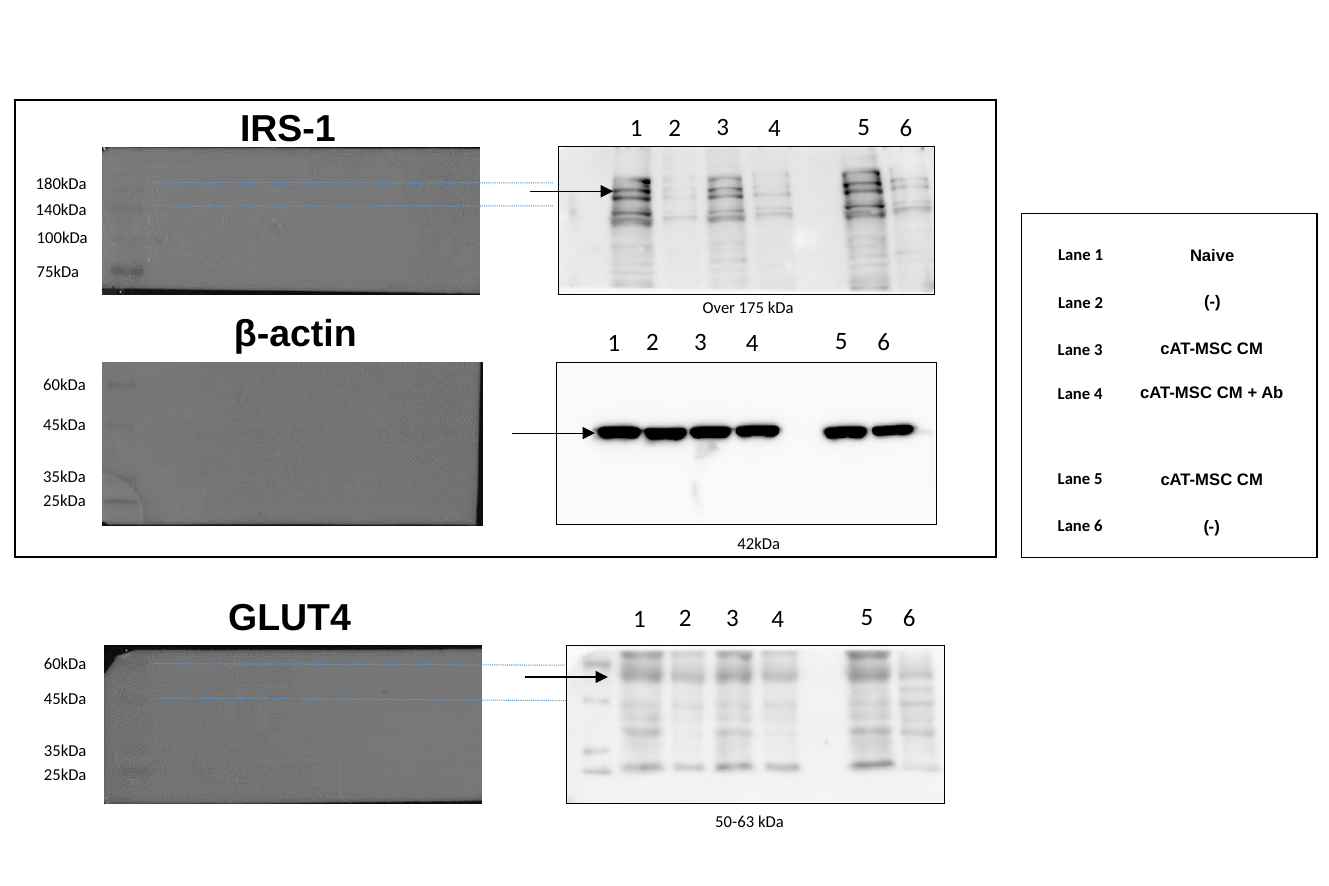

IRS-1
5
3
6
2
1
4
180kDa
140kDa
Lane 1
Naive
(-)
Lane 2
cAT-MSC CM
Lane 3
cAT-MSC CM + Ab
Lane 4
Lane 5
cAT-MSC CM
Lane 6
(-)
100kDa
75kDa
Over 175 kDa
β-actin
5
3
6
2
1
4
60kDa
45kDa
35kDa
25kDa
42kDa
GLUT4
5
3
6
2
1
4
60kDa
45kDa
35kDa
25kDa
50-63 kDa
